# Orthotopic Patient-Derived Xenografts of Gastric Cancer to Decipher Drugs Effects on Cancer Stem Cells and Metastatic Dissemination

**DOI:** 10.3390/cancers11040560

**Published:** 2019-04-19

**Authors:** Julie Giraud, Damien Bouriez, Lornella Seeneevassen, Benoit Rousseau, Elodie Sifré, Alban Giese, Francis Mégraud, Philippe Lehours, Pierre Dubus, Caroline Gronnier, Christine Varon

**Affiliations:** 1 INSERM U1053 Bordeaux Research in Translational Oncology, University of Bordeaux, 33076 Bordeaux CEDEX, France; julie.giraud085@gmail.com (J.G.); damienbouriezbt@gmail.com (D.B.); lornella.seeneevassen@u-bordeaux.fr (L.S.); elodie.sifre@u-bordeaux.fr (E.S.); alban.giese@free.fr (A.G.); francis.megraud@chu-bordeaux.fr (F.M.); philippe.lehours@u-bordeaux.fr (P.L.); pierre.dubus@u-bordeaux.fr (P.D.); caroline.gronnier@chu-bordeaux.fr (C.G.); 2 Animal Facility, University of Bordeaux, 33076 Bordeaux CEDEX, France; benoit.rousseau@u-bordeaux.fr; 3 CHU de Bordeaux, 33076 Bordeaux CEDEX, France

**Keywords:** dissemination, metastases, gastric carcinoma, CD44, CSC, tumorsphere, BKM120, PI3K, PDOX

## Abstract

Gastric cancer is the third leading cause of cancer mortality worldwide. Cancer stem cells (CSC) are at the origin of tumor initiation, chemoresistance, and the formation of metastases. However, there is a lack of mouse models enabling the study of the metastatic process in gastric adenocarcinoma (GC). The aims of this study were to develop original mouse models of patient-derived primary GC orthotopic xenografts (PDOX) allowing the development of distant metastases as preclinical models to study the anti-metastatic efficiency of drugs such as the phosphatidylinositol 3-kinase (PI3K) inhibitor Buparlisib (BKM120). Luciferase-encoding cells generated from primary GC were injected into the stomach wall of immunocompromised mice; gastric tumor and metastases development were followed by bioluminescence imaging. The anti-CSC properties of BKM120 were evaluated on the GC cells’ phenotype (CD44 expression) and tumorigenic properties in vitro and in vivo on BKM120-treated mice. After eight weeks, PDOX mice formed tumors in the stomach as well as distant metastases, that were enriched in CSC, in the liver, the lung, and the peritoneal cavity. BKM120 treatment significantly inhibited the CSC properties in vitro and reduced the number of distant metastases in mice. These new preclinical models offer the opportunity to study the anti-metastatic efficiency of new CSC-based therapeutic strategies.

## 1. Introduction

Gastric cancer has a high incidence and represents the third leading cause of cancer deaths worldwide [1]. Most of them are gastric adenocarcinoma (GC), which is the consequence of chronic infection of the gastric mucosa with *Helicobacter pylori (H. pylori)* [2]. GC has a poor clinical outcome, being detected most of the time too late at advanced metastatic stages. GC are highly heterogeneous both at histological and molecular levels. Except for the rare cases overexpressing the epidermal growth factor receptor HER2, which can be treated with Herceptin, there is no targeted therapy to treat GC patients. The histological criteria-based classification of GC has been recently completed by molecular classifications based on sequencing, which are expected to help in proposing targeted therapies [3,4,5,6]. Current treatment for the non-metastatic forms is based on surgery with perioperative conventional chemotherapies for non-cardia gastric cancer and neoadjuvant chemoradiotherapy for cardia gastric cancer [7,8,9]. New treatment options include the use of immune checkpoint inhibitors in resectable GC [10]. For the unresectable metastatic GC cases, the therapeutic options are usually limited to palliative chemotherapy with a five-year survival rate of less than 20% [10,11].

Targeting agents such as antibodies or small molecules against tyrosine kinases have emerged as a treatment option for GC patients. Activation of the phosphatidylinositol 3-kinase (PI3K) pathway is commonly observed in human cancers and is critical for tumor progression and resistance to cytotoxic chemotherapy [12]. This pathway regulates essential cellular functions including cell proliferation, survival, cytoskeleton rearrangement, and metabolism [13]. PI3K signaling is initiated by the activation of tyrosine kinase receptors, including EGFR, HER2, and MET, leading to the activation of the downstream effector protein kinase B (AKT).

In GC, PI3K signaling is up-regulated through diverse mechanisms involving direct mutation through the amplification of *PIK3CA* or the deregulation of key components such as the monoallelic loss of phosphatase and tensin homologue (PTEN) [14,15]. Accumulating evidence indicates that the PI3K/AKT pathway plays a role in epithelial-to-mesenchymal transition (EMT) induction, which is a fundamental process implicated during embryonic development that is considered a key step toward tumor invasion and metastasis [16,17,18,19,20,21].

Using original patient-derived subcutaneous xenografts of GC (PDX) in immunodeficient mice, we previously contributed to demonstrate the existence of a rare subpopulation of gastric cancer stem cells (CSC) being at the origin of tumor initiation, growth, and chemoresistance [22]. We characterized the cell surface receptor CD44 as one of the best markers of the enrichment of gastric CSC among the cells composing the tumor mass [22]. Moreover, we have highlighted that the chronic infection of gastric epithelial cells with *H. pylori* induces an EMT-like transition leading to the emergence of cells with CSC properties and expressing CD44 both in vitro and in vivo in patients’ derived gastric tissues and in mouse models of experimental infection [23,24]. A major limitation of the mouse models of *H. pylori* infection and/or carcinogens-induced gastric carcinogenesis is that they develop only gastric pre-neoplastic lesions with more rarely in situ carcinomas, but never invasive carcinomas or metastases or peritoneal carcinomatosis as in humans. Likewise, the subcutaneous tumors developed in our PDX mouse models recapitulate the heterogeneity of the primary GC of the patients, but they never metastasize to distant organs in mice [22].

The aim of this study was to develop new preclinical mouse models of GC-forming metastases in order to decipher the metastasis formation process and evaluate the anti-metastatic efficiency of a new therapeutic strategy targeting PI3K activity in GC. Here, we established three mouse models of patient-derived orthotopic xenograft of GC (PDOX) and two models of the orthotopic xenograft of GC cell lines that developed distant metastases in the liver, the lung, and the peritoneal cavity, modeling the disease as in patients. Lung and liver metastases over-expressed the CD44 gastric CSC marker, suggesting that CSC may be involved in dissemination and metastases’ initiation and growth. The treatment of GC cells with the pan-class I PI3K inhibitor BKM120 displayed a cytostatic effect in a dose-dependent manner and decreased the capacity of gastric CSC to form tumorspheres in vitro. Finally, we found that, in mice orthotopically xenografted with GC, BKM120 treatment resulted in a stagnation of tumor progression and a limitation of tumor cell dissemination and metastases formation.

## 2. Results

### 2.1. Development of Patient-Derived Orthotopic Xenograft Mouse Models Developing Distant Metastases

**Figure 1:**
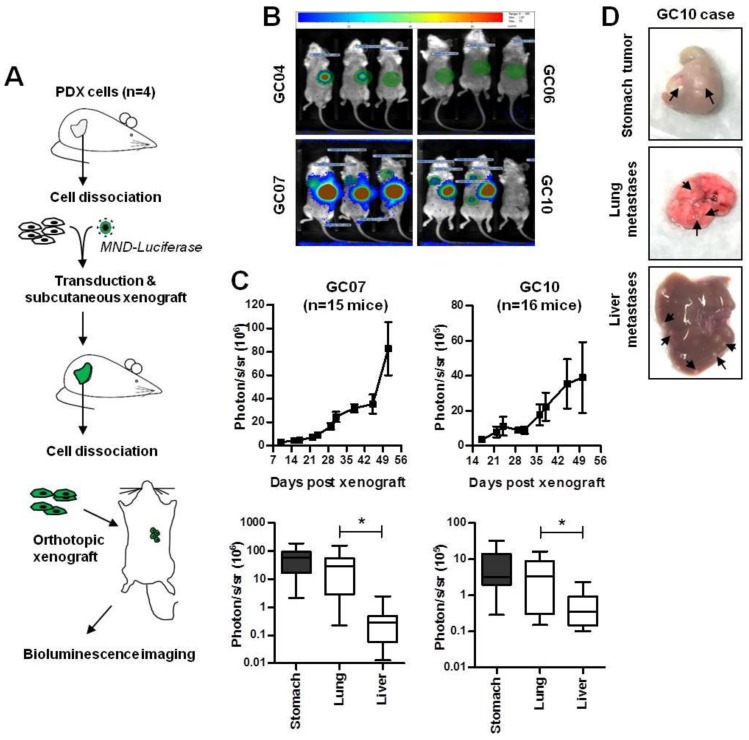
Development of patient-derived orthotopic xenografts generating distant metastases. (**A**) Schematic representation of the injection of patient-derived subcutaneous xenografts of GC (PDX) cells into the subserosa of the stomach of NSG mice. Cells were previously transduced with lentiviruses encoding the luciferase gene (MND-luciferase) and were then amplified subcutaneously before orthotopic xenograft. (**B**) Representative images of bioluminescence imaging on live animals at 6.5 weeks post-orthotopic xenograft. (**C**) Photon quantification of GC07 and GC10 tumor growth on live animals (upper panels) or on recovered organs at end points (lower panels). GC07, *n* = 15 mice; GC10, *n* = 16 mice. (**D**) Representative images of the tumor developed in the stomach, and of macro-metastases developed in the lung and the liver for the GC10 case (pointed out by black arrows). * *p* < 0.05 in one-way ANOVA test.

We previously established several mouse models of PDX, which allowed us to characterize and study the properties of CSC in GC [22,25,26]. These PDX were amplified in mice subcutaneously, and reproduced heterogeneous tumors similar to those of patients, but never metastasized [22]. In order to reproduce the metastatic process, which is at the origin of the low prognosis of GC in humans, we carried out xenografts of these PDX cells directly into the stomach wall. As the engraftment success of primary GC from patients is very low in mice [22], we used four in-house PDX cases that have already been established in mice and well characterized: cases GC04, GC07, and GC10 are of the intestinal histological subtype, while case GC06 is of diffuse type, according to the Lauren histological classification criteria [22]. The GC07 case possesses the advantage of being cultivable in conventional adherent culture conditions conversely to the GC04, GC06, and GC10 cases. First, cells freshly dissociated from PDX tumors were transduced with lentiviruses encoding the luciferase gene, and were thereafter transplanted subcutaneously in NSG mice for amplification. The luciferase-encoding GC cells were recovered from these growing tumors, and 10,000 cells were subsequently xenografted into the subserosa of the stomach wall of new recipient mice (PDOX). The scheme of the experimental procedure is shown in Figure 1A.

In the first GC cells orthotopic xenograft experiment (experiment a, Table 1), tumor growth in the stomach and dissemination in distant organs were followed for up to 25 weeks by bioluminescence imaging on live animals (Figure 1B,C). Bioluminescence imaging was also performed on recovered organs at sacrifice (Figure 1C, bottom panel). PDX cells generated tumors in the stomach and distant metastases in the lung and the liver at variable frequencies according to the cases studied (Table 1), and were macroscopically detectable for some cases (Figure 1D). In this experiment, GC10 and GC07 were the best models of engraftment and dissemination; tumor frequency reached 100% in the stomach for both cases, 75% and 100% frequency in the lung for GC10 and GC07, respectively, and 50% in the liver for GC10 only (Table 1). GC04 generated gastric tumors and lung metastases at a lower frequency than GC10 and GC07, and no liver metastases even after 13 weeks post-xenograft. Surprisingly the diffuse case GC06, which usually forms tumors after three to four weeks post-subcutaneous engraftment, did not grow in the stomach during the 25-week follow-up period, and did not develop distant metastases. As GC10 and GC07 were the best models of engraftment in the stomach, leading to tumor growth and the development of distant metastases, a second orthotopic xenograft experiment was done with only these two cases and with more mice per GC case, which confirmed these results (experiment b, Table 1). Combined together, these experiments showed that GC10 and GC07 are good pre-clinical models to study tumor growth in the stomach and the formation of distant metastases, with tumor development for GC10 and GC07 respectively reaching 94.7% and 100% in the stomach, 73.7% and 100% in the lung, and 79% and 19% in the liver (*n* = 19 mice for GC10 and *n* = 16 mice for GC07 when combining both experiments, as shown in Table 1).

At the end point, photon emission was analyzed in individually recovered organs and showed that the detection of metastatic cells was significantly more important in the lung than in the liver for both the GC07 and GC10 cases (Figure 1C, bottom panel). We also reported the detection of peritoneal tumors for the GC10 case and more rarely for the GC04 and GC07 cases, which developed locally in the case of perforative primary tumors, and were usually associated to tumor detection in the spleen and the pancreas (Table 1).

**Figure 2:**
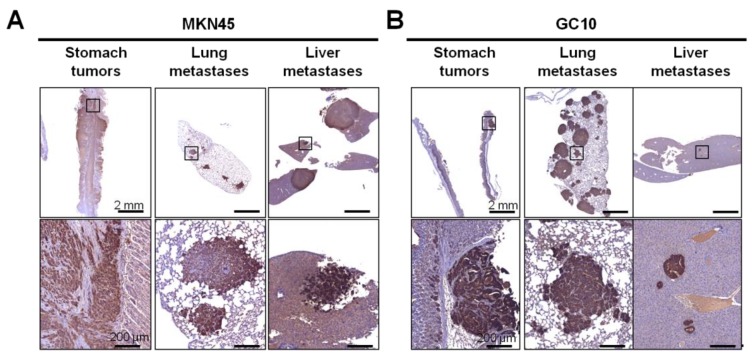
Detection of gastric tumors and distant micrometastases and macrometastases by immunohistochemistry in orthotopic xenograft mice. Representative images of HLA detection by immunohistochemistry on stomach tumors and lung and liver metastases formed with MKN45 cells (**A**) and GC10 PDX case (**B**) eight and 10 weeks after orthotopic xenograft, respectively. The enlarged pictures in the lower panel show the HLA-positive gastric adenocarcinoma (GC) cells in the stomach, lung, and liver metastases. Scale bars: 2 mm in the upper panel, 200 µm in the lower panel.

To increase the number of cases, we realized complementary experiments using three GC cell lines: one is of diffuse type (MKN45) and two are of intestinal type (MKN74 and NCI-N87). After eight to 10 weeks post-orthotopic xenograft, tumor development was determined by macroscopic observation followed by histological analyses of the expression of the human leukocyte antigen (HLA) class-I molecules, which are ubiquitously expressed in human epithelial cells (Figure 2). MKN45 and MKN74 developed tumors in the stomach and distant metastases in the lung with a frequency of 100%. MKN45 and MKN74 also developed metastases in the liver and the peritoneal cavity with a frequency ranging from 50% to 80% (Table 2). On the contrary, only three out of five mice that were xenografted with NCI-N87 cells developed a tumor in the stomach, and no distant metastases were detected up to 10 weeks post-xenograft (Table 2).

In order to determine at which time post-engraftment the GC cells escaped from the stomach to disseminate and initiate distant metastases, we performed experiments of partial resection of the stomach at days 7 and 14 post-orthotopic xenograft of MKN45 luciferase-expressing cells to remove the primary tumor (Supplementary Figure S1). When the resection was performed at day 14 post-xenograft, the mice still developed metastases. However, resection performed at day 7 post-xenograft impaired the development of metastases compared to the non-resected control mouse after 10 days and until 52 days of follow-up (Supplementary Figure S1). Nevertheless, one resected mouse finally developed metastases and tumor in the residual stomach after 28 days, suggesting either an incomplete resection of the primary tumor or circulating tumor cells self-seeding at the primary site, as previously described in breast cancer [27]. These experiments suggest that metastases may arise from the dissemination of GC cells from the primary tumor in the stomach during the first week following orthotopic xenograft.

### 2.2. Metastases Are Enriched in Cancer Stem Cells

**Figure 3:**
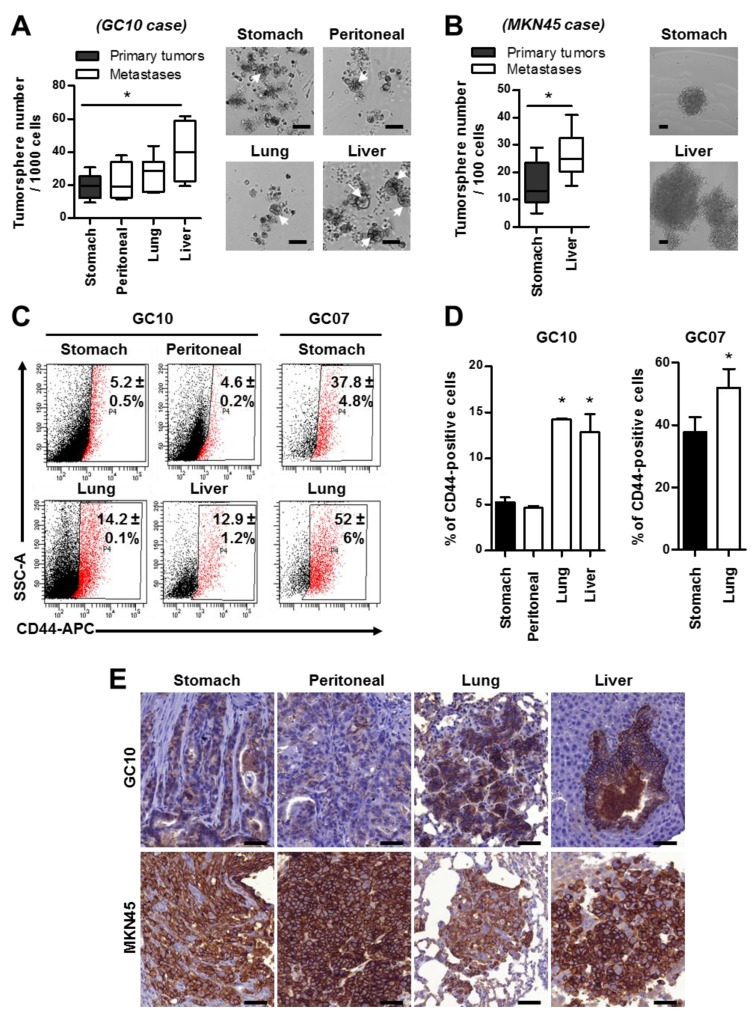
Metastases are enriched in cancer stem cells (CSC). (**A**,**B**) Quantification (left panel) and representative images (right panel) of the tumorspheres formed in vitro by GC10 CSC (**A**) and MKN45 CSC (**B**) derived from primary tumors of the stomach and distant metastases (peritoneal, lung, and liver). The arrows indicate tumorspheres. Data represent the min to max; *n* ≥ 3 mice with nine replicates per experiment and per mice. * *p* < 0.05 in one-way ANOVA and Student’s *t*-test. Scale bars, 50 µm. (**C**,**D**) Flow cytometry dot plots (**C**) and quantification (**D**) of the percentage of CD44-positive cells in primary stomach tumors and metastases derived from GC10 and GC07 cases. Data represent mean ± SEM. of *n* ≥ 3 mice in Kruskal–Wallis and Student’s *t*-test. (**E**) Representative images of CD44 expression detected by immunohistochemistry on tissue sections of stomach tumors and peritoneal, lung, and liver metastases obtained in GC10 and MKN45 orthotopically xenografted mice. Scale bars: 50 µm.

The literature suggests that the CSC subpopulation of tumor cells is not only responsible for tumor initiation, growth, chemoresistance, and tumor heterogeneity, but also for tumor dissemination and metastases formation [28]. To evaluate this hypothesis, we analyzed the proportion of CSC in the metastases and gastric tumors of the orthotopic xenograft models. Tumor cells from stomach tumors and micrometastases formed by GC10 PDOX case and MKN45 cell line were harvested and submitted to the in vitro tumorsphere assay (Figure 3A,B). Only CSC can self-renew and initiate tumor growth in such drastic non-adherent culture conditions [23,25,26]. We observed no differences in tumorsphere formation ability between GC10 cells from the gastric primary tumors and the peritoneal metastases, suggesting the same proportion of CSC in these tumors (Figure 3A). Interestingly, distant metastases, especially those located in the liver, showed higher capacities to initiate tumorspheres than tumors of the stomach (40.5 ± 7.2% versus 19.6 ± 2.6%, respectively) (Figure 3A). The results obtained with this intestinal-type PDX case were confirmed with an opposite case, the diffuse type MKN45 GC cell line, highlighting that the histological subtype and the origin of the GC cells are not at the base of these results. In the MKN45 orthotopic xenograft model, cells from liver metastases formed more tumorspheres than cells from stomach tumors (26.2 ± 1.2% for liver metastases compared to 15.3 ± 1.6% for stomach tumors) (Figure 3B). The expression of CD44, which is one of the best markers of gastric CSC [22], was then analyzed (Figure 3C–E). Flow cytometry analyses showed a significant increase in the percentage of CD44-positive cells in lung metastases compared to the stomach primary tumors for both GC07 and GC10 PDOX cases (Figure 3C,D). Liver metastases formed by GC10 cells also contained significantly more CD44-positive cells than stomach tumors. These results were confirmed by immunohistochemistry analyses on GC10 gastric tumors and metastases (Figure 3E, upper panels). For the MKN45 cell line, in which CD44 expression is very high [22,25], immunohistochemistry analyses revealed a high expression of CD44 in most of the cells in the stomach primary tumors, peritoneal tumors, and lung and liver metastases (Figure 3E, bottom panels). Altogether, these results show that the metastases formed in PDOX models are enriched in CSC.

### 2.3. BKM120 Inhibits GC Proliferation and Decreases the Cancer Stem Cell Population and Properties

**Figure 4:**
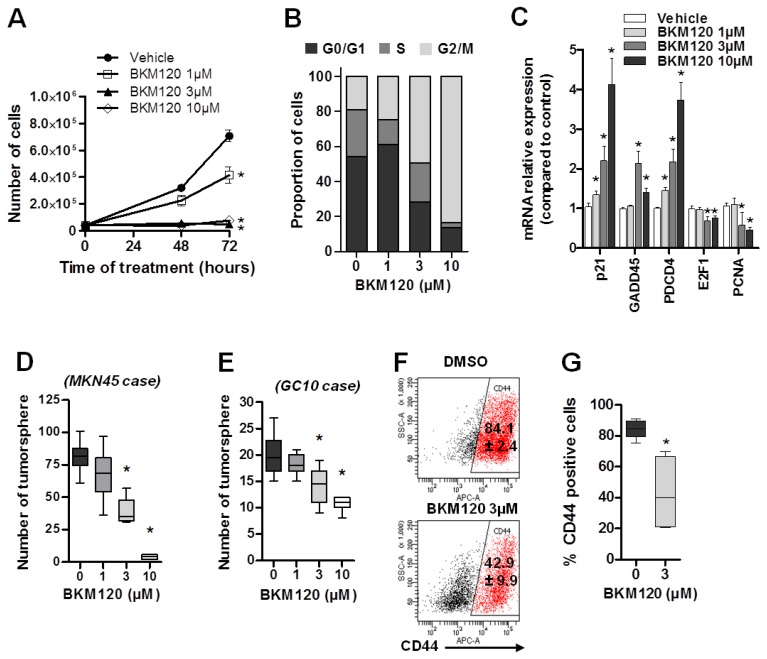
Buparlisib (BKM120) inhibits GC cell proliferation and decreases CSC population and tumorigenic properties. Adherent cells were treated with increasing concentrations of BKM120 or with DMSO as control (vehicle) for 48 h and viability assay (**A**), flow cytometry cell cycle assay (**B**), and relative mRNA expression levels of genes controlling cell-cycle progression (**C**) were performed. *n* = three independent experiments. * *p* < 0.05 compared to control, one-way ANOVA test. MKN45 (**D**) and GC10 (**E**) cells were cultured under non-adherent culture conditions with 1 to 10 µmol/L BKM120 or DMSO for seven days, and the number of tumorspheres formed by 200 or 1000 seeded cells per well, respectively, were quantified (**D**,**E**). Data represent the min to max. *n* = three independent experiments with nine replicates per condition. * *p* < 0.05 in one-way ANOVA test. Cells dissociated from MKN45-treated tumorspheres were stained with anti-CD44-APC antibodies and analyzed by flow cytometry (**F**,**G**). (**F**) Representative dot plot images of flow cytometry analyses and (**G**) quantification of the percentage of CD44-positive cells. Values indicate the mean ± SEM of CD44-positive cells in three independent experiments each performed in duplicates. * *p* < 0.05 vs. control (DMSO alone), Mann–Whitney test.

The PI3K pathway is involved in cell proliferation, apoptosis, cytoskeletal rearrangement, and metabolism [13]. Buparlisib (BKM120) is a pan-class I PI3K inhibitor that has shown an efficiency regarding the proliferation on GC cell lines in vitro [29,30,31]. As expected, BKM120 inhibited in a dose-dependent manner the phosphorylation of serine 473 of its downstream target, AKT (P-AKT^Ser473^), which validates the inhibitory effect of BKM120 on PI3K activity and its downstream targets (Supplementary Figure S2). Moreover, as previously reported [31], we observed a dose-dependent cell proliferation arrest of BKM120-treated MKN45 cells (Figure 4A). Cell cycle analyses revealed that the percentage of cells in G0/G1 phase decreased from 54% (control DMSO) to 28% and 13% in cells treated with 3 µmol/L and 10 µmol/L of BKM120, respectively (Figure 4B). An inversely proportional blocking in G2/M phase was observed in BKM120-treated cells (Figure 4B). This was associated to an increased expression of the cyclin-dependent kinase inhibitor p21, the DNA damage-inducible protein GADD45A, and the programmed cell death protein 4 (PDCD4) (Figure 4C), which are involved in cell cycle arrest. On the contrary, BKM120 reduces the expression of the proliferating cell nuclear antigen PCNA and the transcription factor E2F1 involved in cell cycle progression (Figure 4C).

Then, the effect of BKM120 was evaluated on the CSC subpopulation of GC. The properties of CSC to form tumorspheres and their expression of the CSC marker CD44 were evaluated in vitro in the non-adherent culture conditions of the tumorsphere assay. To overcome a potential bias related to the cytostatic effect of BKM120, we quantified both the ability of cells to form tumorspheres (Figure 4D,E), but also the proportion of CD44-positive cells in the residual tumorspheres (Figure 4F,G). The addition of BKM120 to the culture medium during cell seeding prevented the formation of MKN45 and GC10 tumorspheres in a dose-dependent manner (Figure 4D,E, respectively). MKN45 tumorsphere formation was particularly affected, with the number of tumorspheres decreasing from 81.1 ± 4.1% in the control DMSO-treated condition, to 67.2 ± 6%, 38.9 ± 3%, and 4.1 ± 0.5% at 1 µmol/L, 3 µmol/L, and 10 µmol/L of BKM120, respectively (Figure 4D). More importantly, at ~IC_50_ corresponding to 3 µmol/L of BKM120, the proportion of CD44-positive cells decreased by ~twofold (Figure 4F,G) (84.1 ± 2.5% in control DMSO; 42.9 ± 9.8% in BKM120-treated tumorspheres). Altogether, these results demonstrate for the first time that treating GC cells with the pan-class I PI3K inhibitor BKM120 displays cytostatic effect in adherent conditions and decreased the number of CSC and their tumorigenic properties in vitro in a dose-dependent manner.

### 2.4. BKM120 Limits Tumor Growth and Dissemination In Vivo

**Figure 5:**
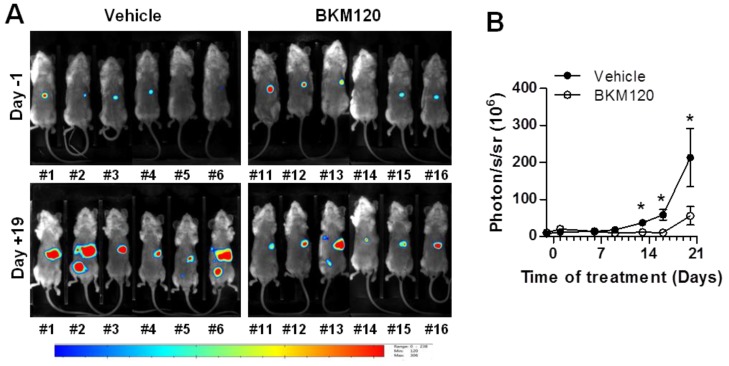
Phosphatidylinositol 3-kinase (PI3K) inhibitor BKM120 limits MKN45 tumor growth in the stomach and metastases formation in vivo. Luciferase-encoding MKN45 cells were xenografted into the stomach of NSG mice. After seven days, mice were randomized and received, one day after, 20 mg/kg BKM120 or vehicle once per day on a schedule of five days on, two days off for three weeks. Tumor development on whole animals was monitored twice a week by in vivo bioluminescence imaging. Representative images of bioluminescence imaging on live mice (**A**) one day before treatment (upper panels) and after 19 days of treatment (bottom panels) and quantification (**B**). 8 ≤ *n* ≤ 9 mice. * *p* < 0.05 vs. vehicle in Student’s *t*-test. #, mouse identification number.

To further investigate the effect of BKM120 on tumor growth and metastatic process in vivo, MKN45 orthotopically xenografted mice were treated with BKM120. Daily oral gavages started eight days after the xenograft of MKN45 cells into the stomach, when tumors became detectable in the stomach, but before metastasis detection by bioluminescence imaging (Figure 5A). One day before the first treatment, mice were randomized into two groups based on the level of photon emission by gastric tumor cells (Figure 5A, upper panels).

In the control group, the growth of gastric tumors increased continuously and significantly from nine days of treatment with the vehicle, whereas no significant increase in gastric tumor growth was observed in the BKM120-treated group for up to 17 days of treatment (Figure 5B). At the end point, corresponding to three weeks of treatment, the group of BKM120-treated mice presented significantly ~3.8-fold less bioluminescent signals in the whole animal compared to the vehicle-treated control group (55.9 ± 24.8 versus 213.0 ± 78.4 respectively, *p* < 0.05) (Figure 5B). Importantly, tumor cells grew in the stomach of all the animals, but the frequency of mice bearing metastases in the lung, the liver, the spleen, and the pancreas was lower in the BKM120-treated mice compared to the vehicle-treated controls (Table 3). BKM120 treatment reduced the frequency of metastases by 12.5% in the lung (7/9 vs. 9/9), 27.8% in the liver (4/8 vs. 7/9), and 41.6% in the spleen and pancreas (2/8 vs. 6/9). Altogether, these results showed that BKM120 limits tumor growth, dissemination, and metastases formation in vivo.

## 3. Discussion

Mouse models of *H. pylori* infection and/or carcinogens-induced gastric carcinogenesis do not develop invasive and metastatic GC as in humans. The best model to study the efficiency of new therapeutic strategies on primary GC and CSC remains PDX mouse models, with the limitation that tumors growing subcutaneously do not metastasize [22,32]. Furukawa et al. pioneered the PDOX nude mouse model with an orthotopic implant using the intact tissue of GC maintained by serial transplantation [33,34]. This technique using tissue samples has the disadvantage of not controlling the number and the viability of the tumor cells present within the grafted tissue. Then, this experiment was reproduced using GC cell lines, which makes it possible to control the number and viability of grafted tumor cells, but reflects the heterogeneity of patients’ tumors less [35,36]. Here, we generated orthotopic injections of suspensions of PDX-derived cells transduced with the luciferase-encoding gene in NSG mice. Tumor growth was followed by in vivo bioluminescence imaging, as previously reported with GC cell lines [37,38]. Experiments resulted in primary tumor growth in the stomach, which was followed in three cases by the dissemination and formation of distant metastases in the same location as in humans (peritoneal cavity and liver), as well as in the lung, which is rare in humans for GC. Metastases in the liver occurred in 79% of NSG mice xenografted with GC10 and in 19% of mice xenografted with GC07. We also showed that not all PDX and GC cell lines can grow in the gastric subserosa and disseminate to form metastases. The diffuse GC06 case did not grow in the orthotopic xenograft experiments, although we previously showed that it grows and develops tumors after subcutaneous xenograft in the same way and at a similar kinetic as GC07 and GC10 cases [22]. In addition, the percentage of CD44+ cells in GC06 is high, reaching about 50% of the tumor cells, as previously reported [22,25]. Thus, its inability to form gastric tumors and metastases cannot be attributed to its diffuse, mesenchymal-like and CSC-enriched phenotype. Moreover, the diffuse GC cell line MKN45 developed stomach tumors and metastases, reinforcing the hypothesis that the histological subtype of GC is not directly responsible for their ability to grow and metastasize in the stomach or not. Thus, we hypothesized that the capacity of GC06 to grow could be linked to its dependency on certain extrinsic factors of the microenvironment in this immunodeficient mouse model, allowing its growth subcutaneously but not in the stomach. In our experiments, the NCI-N87 cell line generated tumors in the stomach in 60% of cases, but did not metastasize. However, Feng et al. reported lymph node and liver metastases in mice injected with 5 × 10^6^ NCI-N87 cells [38]. We can hypothesize that a high number of cells during injection may increase tumor growth in the stomach and the frequency of metastatic development, but it could also increase the risk of leakage and unspecific dissemination related to the injection of so many cells. In our experiments, the risk of leakage was limited by the low volume of injection and the low number of injected cells (1 × 10^4^ cells) compared to other studies. In addition, the time-course experiments of gastric tumor resections demonstrated that metastases are initiated during the two first weeks after GC cell xenograft in the stomach. Interestingly, in one of the gastric tumor-resected mice, the presence of tumor cells was observed in the residual stomach at sacrifice. This result should indeed be confirmed in more animals, but suggests either an incomplete tumor resection or the self-seeding of circulating tumor cells (CTC) in the primary niche. Indeed, Kim et al. demonstrated that the dissemination of cells is not unidirectional, and that aggressive CTC in the peripheral blood of patients with breast cancer, colon cancer, and melanoma can colonize their tumors of origin, being at the origin of local recurrence seeded by disseminated cells following ostensibly complete tumor excision [27].

The tumorigenic and invasive properties of CSC are closely associated to their intrinsic and extrinsic plasticity and to EMT. Brabletz et al. first described migrating CSC at the invasive front of tumors [39]. CSC properties, including tumorsphere formation capacity and CD44^high^ expression profiles can be acquired through the induction of EMT in breast tumors [28,40] and in GC, as we have previously shown [41]. In GC, previous studies have reported that CD44-positive cells generate gastric tumors with a higher frequency than CD44-negative cells when implanted in the stomach [42]. However, the authors did not evaluate the development of metastases in these animals. Moreover, GC patients having CD44-positive CTC are more prone to develop metastases and recurrence than patients having CD44-negative CTC [43,44]. In agreement with these studies, we show here that the proportion of CSC is increased in metastases compared to the gastric tumors formed by GC cell lines and patient-derived GC cases, suggesting that CSC may drive CTC dissemination and the formation of metastases. Further studies are required to fully characterize the subpopulation of CSC-derived CTC that is able to initiate metastases and may be self-seeding following surgical resection of the primary gastric tumor.

We previously showed that targeting the AMP-activated protein kinase / mammalian target of rapamycin (AMPK/mTOR) pathway, downstream of PI3K signaling, with metformin decreased the CSC population and their tumorigenic properties in GC [26]. The PI3K pathway controls cell proliferation, apoptosis, cytoskeletal rearrangement, and metabolism [13], and is being more and more linked to CSC maintenance and plasticity. Interestingly, in the molecular classification of Lei et al. distinguishing GC into proliferative, metabolic, and mesenchymal subtypes [6], tumors of the mesenchymal phenotype, which are enriched in cells with features of CSCs, are particularly sensitive to PI3K/AKT/mTOR inhibitors in vitro [6]. BKM120 is a pan-class I PI3K inhibitor, acting as a competitive ATP inhibitor targeting the catalytic activity of p110α, β, δ, and γ isoforms with the same affinity, and exhibiting its activity with or without mutation in the PIK3CA. BKM120 has shown an efficiency alone or in combination with other chemotherapies in several phase I and phase II clinical trials in patients with advanced and refractory solid tumors [45,46,47], advanced leukemia [48], ovarian and breast cancer [49], endometrial carcinoma [50], prostate cancer [51], head and neck squamous cell carcinoma [52], and in breast cancer with phase III clinical trials [53]. No clinical trials have been published concerning BKM120 in GC patients, and only three articles have shown that BKM120 inhibits the proliferation on GC cell lines in vitro [29,30,31]. In GC patients, AKT^Ser-473^ phosphorylation, which is a direct downstream target of PI3K, is correlated with tumor progression and a poor clinical outcome [54]. In this study, we confirmed that BKM120 treatment reduced GC cell proliferation in vitro and strongly inhibited P-AKT^Ser473^. More originally, we show for the first time that BKM120 decreased the population of CD44-positive CSC and their tumorigenic properties in vitro both in GC cell lines and more importantly in patient-derived GC cases. Using the original orthotopic xenograft models that we developed, we showed that BKM120 treatment in vivo led to a significant reduction of tumor growth and a reduction in the number of metastases-bearing mice. On a more basic point of view, the resection experiments carried out before the metastasis initiation period further validated our orthotopic xenograft models as pertinent for evaluating CSC-based anti-tumorigenic and/or anti-invasive therapeutic strategies.

## 4. Materials and Methods

### 4.1. Cancer Cell Lines and Patient-Derived Xenograft Cell Culture

All the cell lines were mycoplasma-free (verified by PCR). MKN45 and MKN74 gastric cancer cell lines, authenticated by short tandem repeats profiling, were cultured in RPMI 1640-Glutamax supplemented with 10% heat-inactivated fetal bovine serum (10% FBS) and 50 U/mL of penicillin and 50 µg/mL of streptomycin (1:100 P/S) at 37 °C in a 5% CO_2_ humidified incubator (all from Thermo Fisher Scientific, Villebon sur Yvette, France). The NCI-N87 cell line was cultured in DMEM F12-Glutamax supplemented with 10% FBS and 1:100 P/S. Cell viability was measured using trypan blue dye exclusion 48 h and 72 h after treatment. GC04, GC06, GC07, and GC10 are patient-derived GC cells successfully established by serial subcutaneous xenografts in non obese diabetous/severe combined immunodeficiency/interleukin-2Rγ null (NSG) mice, as previously described [22]. The status for Epstein-Barr Virus (EBV) was determined by molecular methods on tumor tissue sections (in situ hybridization) and on DNA extracted from PDX tumors according to standard procedures of the Haut-Lévèque hospital of University Hospital Center of Bordeaux; all GC cases were EBV-negative. Mutations have been searched using the NGS Colon and Lung Panel for KRAS, EGFR, BRAF, PI3KCA, AKT1, ERBB2, PTEN, NRAS, STK11, MAP2K1, ALK, DDR2, CTNNB1, MET, TP53, SMAD4, FBX7, FGFR3, NOTCH1, ERBB4, FGFR1, and FGFR2. GC04 is PIK3CA mutated (c.1633G > A p. (Glu545Lys)), GC06 is KRAS mutated (c.175G > A p. (Ala59Thr) and c.38G > A p. (Gly13Asp)), GC07 is mutated for STK11 (c.1019A > G p. (Tyr340Cys)) and GC10 is mutated for TP53 (c.818G > A p. (Arg273His)). When the tumor size reached between 200–400 mm^3^, tumors were recovered and dissociated with a human tumor dissociation kit in C tubes and a GentleMACS dissociator following manufacturer recommendations (all from MACS, Miltenyi, Paris, France). Cells were filtered successively though 70 µm and 40 µm cell strainers, and red blood cells were lysed. Then, cells were processed for tumorsphere formation, flow cytometry, and lentiviral transduction, or were xenografted in mice.

### 4.2. Production of Lentiviral Particles and Transduction of Cells

Gene transfer of the luciferase protein was done using a pRRLsin-MND-Luc-WPRE lentiviral vector. Particles were produced by the vectorology platform of Bordeaux University. Dissociated PDX cells were transduced with a multiplicity of infection (MOI) of 30 lentiviral particles per cells overnight in DMEM F12-Glutamax medium containing 10 mM of Hepes in a 5-mL polypropylene tube. Twelve h later, cells were rinsed and prepared for subcutaneous xenograft amplification before orthotopic experiments. For the MKN45 cell line, adherent cells were transduced with pRRLsin-MND-Luc-IRES2-ZsGreen-WPRE lentiviral particles (MOI 30) in DMEM F12-Glutamax medium containing 10 mM of Hepes for 24 h.

### 4.3. Orthotopic Xenograft and Resection Experiments

Five-week-old NSG female mice from the Level 2 animal facility of the University of Bordeaux were maintained in accordance with the institutional guidelines of the institutional animal use and care committee (accreditation number B33-063-916, received on 23 May 2016). All of the animal experiments were approved by the French Ethics Committee on Animal Experiments CEEA50 of Bordeaux (Authorization n°A12005, ref 2017103118319700 v7).

Mice received an intraperitoneal (i.p.) injection of 300 μg/kg buprenorphine (Vetergesic, Centavet, Dinan, France) 30 min before laparotomy. Animals were anesthetized with isoflurane in sterile condition, and a 6-mm incision was made on the right side of the animal to expose the stomach. Then, 10,000 cells from the GC cell lines and PDXs cells, suspended in 30 μL of DMEM F12-Glutamax containing 1% P/S and 10% FCS, were injected into the subserosa using a 500-µL syringe supplied by a 30-gauge needle (Terumo, Guyancourt, France) under a dissection microscope. The peritoneal incision and the skin incision were sutured (5.0 Ethicon, Vicryl). The wound was disinfected with a spray of chlortetracycline (Cyclo spray, Centavet, Dinan, France). For the primary tumor resection experiment, cells were suspended in 20 μL of DMEM F12-Glutamax containing 1% P/S and 10% FCS and 7 mg/mL ice-cold matrigel (Ozyme, Montigny-le-Bretonneux, France). Seven and 15 days after orthotopic xenograft, the stomach was exposed by laparotomy and the primary tumor was excised following similar experimental surgical procedures as done during a sleeve gastrectomy.

### 4.4. Bioluminescence Imaging

Mice were monitored for up to 25 weeks by in vivo bioluminescence imaging (Biospace Lab, Nesles-la-Vallée, France) upon i.p. injection of 3.3 mg of D-Luciferin (Promega, Charbonnières-les-Bains, France). At the end point, mice were sacrificed, and organs were macroscopically analyzed and collected for histological analyses. In the luciferase-expressing models, photon emission on recovered organs was measured after their incubation for 5 min in a solution of 3.3 mg/mL D-luciferin.

### 4.5. Immunohistochemistry

Tissues were fixed 16 h in 3.7% formol and processed for paraffin embedding. Then, 3 µm tissue sections were deparaffinized in xylene and rehydrated in graded alcohol. For immunohistochemistry experiments, antigen enhancement was performed on slides in boiled citrate buffer ph6. Primary antibodies human anti-HLA class 1 (1:100e, Abcam 70328, Cambridge, UK) and human anti-CD44 (1:100e, BD Bioscience 550392, Allschwil, Switzerland) were incubated respectively for 1 h and 2 h at room temperature (RT). Then, slides were incubated with Avidin/Biotin blocking reagent (SP-2001, Vector Laboratories, Burlingame, CA, USA) followed by successive incubations of biotinylated horse anti-mouse Immunoglobulin G incubation and VECTASTAIN^®^ Elite^®^ ABC HRP reagent according to manufacturer’ recommendations (Vector Laboratories, Burlingame, CA, USA). Immunolabeling was revealed in liquid substrate diaminobenzidine chromogen (DAKO). Slides were counterstained with hematoxylin, dehydrated, and mounted with an Eukitt-mounting medium. Slides were scanned with Pannoramic Scan (3D Histech, Budapest, Hungary).

### 4.6. PI3K Inhibitor Preparation and Treatments

BKM120, a pan-class I PI3K inhibitor, was purchased from Sigma-Aldrich (Saint-Quentin Fallavier, France). For the in vitro experiments, BKM120 was prepared as 10 mmol/L stock solution in DMSO and conserved at −20 °C for ≤2 months. Adherent cultured cells were treated for 48 h to 72 h one day after seeding with a medium change. Three-dimensional cultured cells were treated during the seeding of the cells to analyze tumorsphere formation. Tumorspheres were treated 4 days after seeding to analyze CD44 expression. For the in vivo experiments, BKM120 was dissolved in 10% NMP (1-methyl-2-pyrrolidone) and 90% PEG300 (all from Sigma-Aldrich). Vehicle treatment consisted of a solution containing 10% NMP and 90% PEG300. Solution was administrated by oral gavage at 20 mg/kg once per day on a schedule of five days on, two days off schedule for three weeks. The GC10 PDX cell and MKN45 gastric cancer cell lines treated with BKM120 were PIK3CA wild-type.

### 4.7. Tumorsphere Culture and Quantification

MKN45 cells were cultured in serum-free DMEM F12-Glutamax medium containing 1:100 P/S, 0.3% glucose, N2 supplement (all from Thermo Fisher Scientific), 20 ng/mL of human epidermal growth factor, 20 ng/mL human basic growth factor and insulin 5 µg/mL (Sigma-Aldrich) onto non-adherent 96-well culture plates (previously coated with a 10% poly-2-hydroxyethyl methacrylate (Sigma-Aldrich) solution in 95% (*v/v*) ethanol and dried overnight at 56 °C). PDX cells (GC07 and GC10) were cultured in tumorsphere medium supplemented with 2% FBS. The formed tumorspheres, ≥50 µm, were counted 7 days post-seeding and treatment with BKM120. Images of tumorspheres were taken using a ZOE^TM^ Fluorescent Cell Imager (Bio-Rad, Irvine, CA, USA).

### 4.8. Flow Cytometry and Cell Cycle Assay

MKN45 were grown for 4 days in non-adherent conditions and were then treated with 3 µmol/L of BKM120 or DMSO (control) for 48 h. Tumorspheres were dissociated with Trypsin- Ethylenediaminetetraacetic acid (EDTA) and rinsed with ice cold-buffer containing 2 mM of EDTA and 0.5% bovine serum albumin (BSA) in PBS. Cells were stained with 1:25 CD44-APC antibodies (G44-26 clone, BD Biosciences) or isotype controls for 25 min at 4 °C. Cells were rinsed three times with cold buffer and were processed for flow cytometry. Dead cells were excluded based on side scatter analysis and 7-Aminoactinomycin D (50 µg/mL, BD Pharmingen, 559925) negative staining. For cell cycle analysis, 24-h adherent cells were treated for 48 h with increased concentrations of BKM120 and DMSO as control. Cells were dissociated by trypsinization and were stained for 1 h in cold fluorochrome solution containing 50 µg/mL of propidium iodide, 0.1% (*w/v*) sodium citrate and 0.1% triton (*v/v*). Flow cytometry was performed using a BD FACSCanto II instrument and DIVA software (BD Biosciences).

### 4.9. RNA Extraction and RT-qPCR

Total RNA was extracted with TRIzol^TM^ reagent (Thermo Fisher Scientific) following the manufacturer’s instructions. Reverse transcription was performed using the Quantitech Reverse Transcription kit (QIagen, Venlo, The Netherlands) following the protocol provided from the manufacturer. Real-time PCR was performed using the SYBR-qPCR-Premix Ex-Taq (Takara, Shiga, Japan) and 0.3 µmol/L of specific primers (Supplementary Table S1). Amplified RNA samples were calculated using the 2^−∆∆CT^ method with HPRT1 and TBP as normalizers.

### 4.10. Protein Isolation and Western Blotting

Treated and control whole cells (48 h) were washed twice with cold PBS and lysed in RIPA buffer containing a cocktail of protease inhibitor (P8340), phosphatase inhibitors (P0044 and P5726), and 1 mM of phenylmethylsulfonyl fluoride supplemented with 5 mmol/L of EDTA (all from Sigma-Aldrich). The lysate was centrifuged (13,000 rpm, 15 min at 4 °C), and the supernatant was stored at −80 °C until further use. Total proteins (25 μg) were electrophoresed by an 8% sodium dodecyl sulfate polyacrylamide gel electrophoresis (SDS-PAGE) and transferred onto nitrocellulose membranes. The membranes were blocked in 5% BSA in PBS-Tween 20 0.1% and probed overnight at 4 °C with primary antibodies to P-AKT^ser473^ (CST 4060), AKT (CST 4685) and GAPDH (Sc-25778). Then, IRDye 800-conjugated goat anti-rabbit secondary antibody (LI-COR, Inc., Lincoln, NE, USA) was diluted 1:5000 and probed for 60 min at room temperature. Antibody-bound proteins were detected using ChemiDoc™ Imaging Systems (Bio-Rad).

### 4.11. Statistical Analysis

The results are expressed as the mean ± SEM of three independent experiments. Statistical tests were performed using GraphPad Prism Version 5.0 (GraphPad, San Diego, CA, USA), using Mann–Whitney, Student’s *t*-test or ANOVA with Bonferroni as the post-hoc test and the Kruskal–Wallis test for multiparametric comparisons. A 5% cutoff was used to validate the result significance.

## 5. Conclusions

Using these new models, we demonstrated that BKM120 treatment targets gastric CSCs and leads to a significant reduction in the number of metastases-bearing mice. The development of these new preclinical models offers the opportunity to decipher the basic mechanisms of CSC dissemination and study the efficiency of drugs targeting gastric CSC to block their growth and, most importantly, their metastatic spread, which is responsible for most of the GC-related deaths. These models could also be useful to further study and characterize the subpopulation of CSC-derived CTC initiating metastases, and their putative self-seeding mechanisms following surgical resection of the primary gastric tumor.

## Figures and Tables

**Table 1:** Number of tumor-bearing mice per total of NSG mice implanted with luciferase-encoding gastric PDX cells into gastric subserosa in two independent experiments.

Number of Tumor-Bearing Mice ^1^/Number of Xenografted Mice						
Experiment	Case	Stomach	Lung	Liver	Peritoneal Tumors	Weeks
a	GC04	3/4	2/4	0/4	1/4	13
b	GC06 GC07 GC10 GC07 GC10	0/4 4/4 4/4 12/12 14/15	0/4 4/4 3/4 12/12 11/15	0/4 0/4 2/4 3/12 13/15	0/4 0/4 0/4 1/12 5/15	25 8 10 8 8

^1^ Tumor development was monitored by bioluminescence imaging until indicated weeks.

**Table 2:** Number of tumor-bearing mice per total of NSG mice implanted with gastric cancer cell lines into gastric subserosa.

Number of Tumor-Bearing Mice ^1^/Number of Xenograft Mice					
Case	Stomach	Lung	Liver	Peritoneal Tumor	Weeks
MKN45 MKN74 NCI-N87	5/5 4/4 3/5	5/5 4/4 0/4	4/5 2/4 0/5	3/5 2/4 0/5	8 8 8–10

^1^ Tumor development was examined macroscopically after sacrifice and by histological analyses of human-specific HLA expression.

**Table 3:** Number of tumor-bearing mice per total NSG mice implanted with MKN45 and treated with BKM120 and vehicle (DMSO-treated mice).

Number of Tumor-Bearing Mice ^1^/Number of Xenograft Mice				
Case	Stomach	Lung	Liver	Spleen and Pancreas
Vehicle BKM120	9/9 8/8	9/9 7/8	7/9 4/8	6/9 2/8

^1^ Tumor development was determined by photon emission on organs at end point.

## Supplementary Materials

The following are available online at https://www.mdpi.com/2072-6694/11/4/560/s1. Figure S1: Metastasis follow-up after primary tumor resection, Figure S2: Validation of BKM120 inhibition on AKT Ser473-phosphorylation, Table S1: List of primers used for RT-qPCR analysis.
