# Supplementary material for: Orthotopic Patient-Derived Xenografts of Gastric Cancer to Decipher Drugs Effects on Cancer Stem Cells and Metastatic Dissemination
Source: Cancers (Basel). 2019 Apr 19;11(4):560. doi: 10.3390/cancers11040560 (PMC6520896; doi:10.3390/cancers11040560)
Supplement: Supplementary file 1 [file cancers-11-00560-s001.pdf]

Supplementary Materials: Orthotopic Patient-Derived Xenografts of Gastric Cancer to Decipher Drugs Effects on Cancer Stem Cells and Metastatic Dissemination

Julie Giraud, Damien Bouriez, Lornella Seeneevassen, Benoit Rousseau, Elodie Sifré, Alban Giese, Francis Mégraud, Philippe Lehours, Pierre Dubus, Caroline Gronnier and Christine Varon

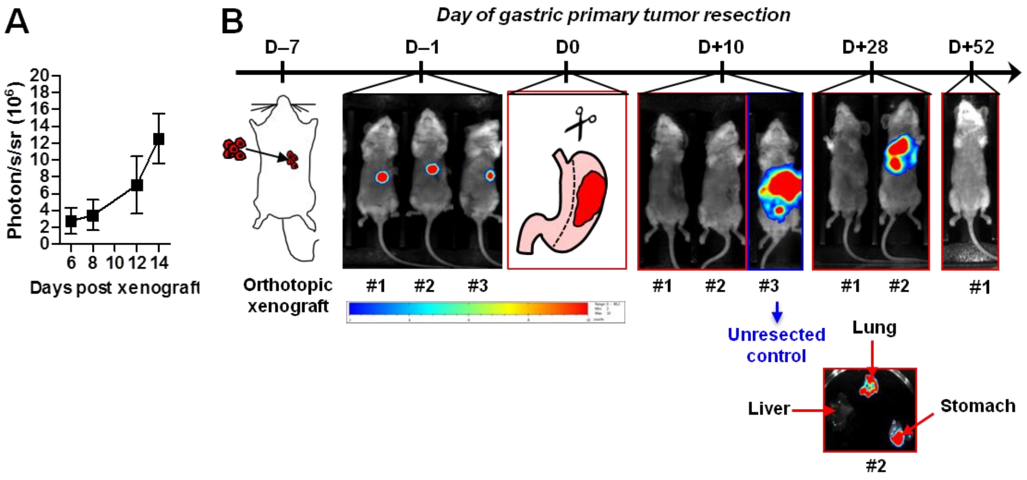

Figure S1. Metastasis follow-up after primary tumor resection. MKN45 cells expressing luciferase were xenografted into the sub-serosa of 3 NSG mice. Seven days after xenograft, which corresponds to the beginning of tumor growth detected by bioluminescence imaging (**A**), primary tumors were resected in 2 out of 3 mice (**B**). The growth of gastric tumors and metastases was followed in live animals and in organs (lower panel) by bioluminescence imaging up to 52 days. #, mouse identification number.

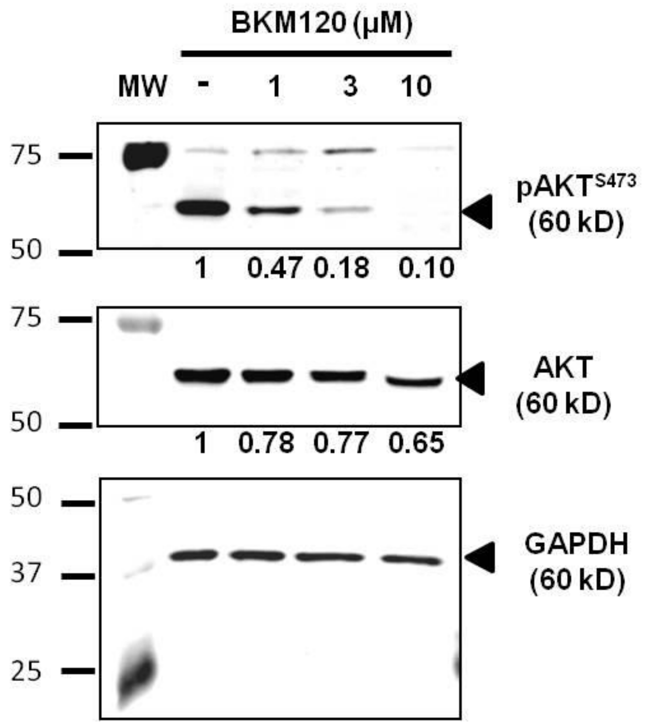

Figure S2. Validation of BKM120 inhibition on AKT Ser473-phosphorylation. Representative western blots of active AKT (pAKT[S473]) and total AKT level with GAPDH as a loading control. MKN45 cells were treated 48 h with increasing concentrations of BKM120 (1, 3 and 10 µM) and DMSO as a control. BKM120 induces a strong dose-dependent inhibition of AKT phosphorylation on Ser473. MW, molecular weight. Numbers indicate the densitometry intensity ratio (AKT or pAKTs473/ GAPDH compared to no BKM120-treated cells) for each band.

Table S1. List of primers used for RT-qPCR analysis.

Gene	Sequence Frame (5′-3′)	Sequence Reverse (5′-3′)
E2F1	GGAACTGAGGCCTGGGTGAT	CCCATGGCTGTCAGTCAGTCT
GADD45	GCAGGATCCTTCCATTGAGA	CTCTTGGAGACCGACGCTG
HPRT1	TGGTCAGGCAGTATAATCCA	GGTCCTTTTCACCAGCAAGCT
P21	CCTCATCCCGTGTTCTCCTTT	GTACCACCCAGCGGACAAGT
PDCD4	CAGTTGGTGGGCCAGTTTATT	AGAAGCACGGTAGCCTTATCCA
PCNA	AGGGCTCCATCCTCAAGAAGG	TGGTGGTTCAAATACTAGCGC
TBP	TGCACAGGAGCCAAGAGTGAA	CACATCACAGCTCCCCACCA

© 2019 by the authors. Licensee MDPI, Basel, Switzerland. This article is an open access article distributed under the terms and conditions of the Creative Commons Attribution (CC BY) license (http://creativecommons.org/licenses/by/4.0/).
